# Ultrasound-Guided Thermal Ablation of Thyroid Nodules: Technicalities Progress and Clinical Applications, Especially in Malignant Thyroid Nodules

**DOI:** 10.3389/fonc.2021.761005

**Published:** 2021-11-17

**Authors:** Enock Adjei Agyekum, Jian-hua Fu, Fei-Ju Xu, Yong-Zhen Ren, Debora Akortia, Qing Chen, Xiao-Qin Qian, Yuguo Wang, Xian Wang

**Affiliations:** ^1^ Department of Ultrasound, Jiangsu University Affiliated People’s Hospital, Zhenjiang, China; ^2^ School of Medicine, Jiangsu University, Zhenjiang, China; ^3^ Department of Interventional Radiology, Jiangsu University Affiliated People’s Hospital, Zhenjiang, China; ^4^ School of Public Health, Kwame Nkrumah University of Science and Technology, Kumasi, Ghana; ^5^ Department of Ultrasound, Nanjing Lishui District Hospital of Traditional Chinese Medicine, Nanjing, China

**Keywords:** thyroid nodule, thermal ablation, thyroidectomy, ultrasonography, thyroid carcinoma

## Abstract

Thyroid nodules are commonly encountered in health care practice. They are usually benign in nature, with few cases being malignant, and their detection has increased in the adult population with the help of ultrasonography. Thyroidectomy or surgery is the first-line treatment and traditional method for thyroid nodules; however, thyroidectomy leaves permanent scars and requires long-term use of levothyroxine after surgery, which makes patients more reticent to accept this treatment. Thermal ablation is a minimally-invasive technique that have been employed in the treatment of benign and malignant thyroid nodules nodules, and have been shown to be effective and safe. Several studies, including long-term, retrospective, and prospective studies, have investigated the use of ablation to treat benign thyroid nodules and malignant thyroid nodules, including papillary thyroid carcinoma. Here, we review the recent progress in thermal ablation techniques for treating benign and malignant nodules, including their technicalities, clinical applications, pitfalls and limitations, and factors that could affect treatment outcomes. Special in-depth elaboration on the recent progress of the application of thermal ablation therapy in malignant thyroid nodules.

## Introduction

Thyroid nodules are a common occurrence in health care practice, and their detection has increased in the adult population with the help of ultrasonography (US) (1). The common risk factor is iodine deficiency (2). Thyroid nodules are usually benign, and few are malignant (3). Most benign thyroid nodules are asymptomatic and/or non-functioning and usually do not require treatment (4). Treatment is necessary in patients with subjective symptoms or cosmetic problems, which are associated with nodular volume (1). In some cases, large or multiple thyroid nodules can cause local pain and difficulties in swallowing (5) due to compression of the esophagus and trachea (2).

According to the 2015 American Thyroid Association guidelines and 2016 Chinese expert consensus and guidelines, thyroidectomy and surgery are the traditional, first-line treatments for benign and malignant thyroid nodules (3). Thyroidectomy leaves permanent scars and requires a long-term use of levothyroxine after surgery, thus patients are less likely to choose this treatment (3). Other complications include reduced thyroid function and permanent injury to the superior or recurrent laryngeal nerves (6). In addition, surgery can present clinically significant morbidities, such as hypoparathyroidism, airway obstruction associated with bilateral vocal dysfunction, and hematoma (7). Recently, to avoid surgery and its potential complications, thermal ablation (TA) for benign and malignant thyroid nodules, including laser ablation (LA), radiofrequency ablation (RFA) and microwave ablation(MWA) have been proposed and widely used (8, 9).

RFA, MWA, and LA have many similarities. For instance, they are minimally invasive (10, 11). Thermal ablation can be employed in the treatment of benign thyroid nodules and malignant thyroid nodules (4, 10, 12). They act by inducing tissue necrosis through heat and they are less expensive and cheaper than traditional methods, such as surgery (13, 14, 15). This paper reviews the current progress of thermal ablation, when used in the treatment and management of benign and malignant thyroid nodules. We consider their technique’s progress, clinical applications, pitfalls and limitations, factors that could affect treatment outcomes, and special in-depth elaboration on the recent progress of the application of thermal ablation therapy in malignant thyroid nodules.

## Ultrasound-Guided Thermal Ablation Techniques and Recent Progress

RFA involves the generation of an electric field by a radiofrequency generator connected to an internally cooled electrode needle that causes frictional agitation at the ionic level, which in turn leads to the generation of heat. RFA devices usually use monopolar radiofrequency electrodes, which have collecting pads, and bipolar devices, which have no pads and shorter electrodes (7–10-cm long). Typically, MWA equipment consists of a microwave generator, a flexible low-loss coaxial cable, and an internally cooled antenna corresponding to a 14G to 16G needle (16, 17). The generator has the ability to produce 1–100 W of power at 2450 MHz in the form of pulse or continuous flow (18), which causes water molecules in the ablated tissue to oscillate and heat tissue to cytotoxic levels, thus causing cell death. Subsequently, the induced coagulative necrosis is degraded by the patient’s own immune system (1). The percutaneous LA method is similar to RFA and MWA, and typically involves the use of a 21G biopsy needle with a fixed power protocol of 3 W; the total energy delivered for each fiber/illumination is usually between 1,200 and 1,800 J (19). For the percutaneous LA method, the stylet of the needle is removed and a plane‐cut optic fiber is moved through the sheath of the needle, after which the distal fiber tip is interstitially positioned into the previously defined portions of the target nodules (20).

Percutaneous thermal ablation is normally performed in an outpatient setting by an experienced physician. Intravenous access is obtained through the antecubital vein, and partial pressure of oxygen, heart rhythm, respiratory rate, and blood pressure of the patient are continuously monitored (21). Patients are normally placed in the supine position with the neck extended, and vital signs are monitored (22, 23). To avoid the occurrence of severe hemorrhage, vessels that are found along the approach route are carefully evaluated using Doppler US  (24). Before ablation, local anesthesia with 1% lidocaine is typically injected at the subcutaneous puncture site and the periphery of thyroid nodules to reduce the pain during the skin incision and insertion of an electrode; in instances where cystic components are present, fluid is aspirated before US-guided percutaneous ablation (23, 25–27).

Some important recent advances in thermal ablation technology are as follows. In the “barrier” technique, first sodium hyaluronate or normal saline is injected between the thyroid capsule and surrounding tissues to completely separate the thyroid capsule and surrounding tissues and form a liquid isolation zone with a width of about 5–10 mm. This helps to avoid thermal damage to the surrounding tissues, particularly larynx injury of the recurrent nerve. Sodium hyaluronate has been employed in thyroid surgeries and can be chosen as the isolation liquid as an alternative to normal saline because it has a greater density; it thus has a good shaping effect and long absorption time (>2 h), which meets the requirements of operation time (28). In addition, sodium hyaluronate can prevent postoperative adhesions. In the “trans-isthmic” puncture technique, an electrode is inserted into the nodule using the trans-isthmic approach (29). This technique has several benefits, including the prevention of electrode movement in case the patient swallows or talks during the ablation process, and allowing the electrode to pass through a sufficient amount of thyroid parenchyma in order to prevent fluid leak outside the thyroid gland and prevention of damage to vital structures and vessels (30). In the “artery-first” ablation technique, it is important to prioritize the ablation of the nourishing artery and block the blood supply of the tumor. The technique can reduce bleeding during ablation, and on the other hand, it can prevent incomplete thermal ablation of the tumor due to the heat sink effect, which is characterized by the presence of nearby vessels, which can dissipate the thermal energy away from target tissues. In the “moving-shot” ablation technique, the electrode tip is first positioned in the deepest and the most remote portion of the nodule, which aids the observation of the electrode tip without disturbances caused by microbubbles (2, 11). Ablation of the thyroid nodules is initiated using a moving-shot technique that usually starts with 10–30 W of power (30, 31). A hyperechoic zone is normally seen within the nodule after 20–45 s, and the electrode is then moved to another portion of the nodule; in instances where there is no occurrence of a hyperechoic zone after 45 s, the power is increased in 5-W steps (32). The ablation time has been reported to range from 2 to 16 min per nodule (mean time, 8 min) (25). Treatment is ceased when all thyroid parenchyma requiring treatment have been replaced by an echogenic gas artefact (33, 34). In the “needle-selection” technique, the size of the nodule, cystic, or solid components and vascularity is taken into consideration when selecting needle types and performing the procedure. For nodules with a volume ≤15 mL, a single fiber LA needle is best used, and for solid nodules with a volume >15 mL, multiple fiber LA, RFA, or MWA ablation needles can be selected. For nodules with both cystic and solid components with a volume >15 mL, the fluid component is first drained, and then the needle can be selected according to the nodular volume after draining (35). In the “Suitable for mass shape” ablation technique, for some irregular mass, multiple needles should be placed in accordance with the shape of the mass to avoid incomplete ablation of the mass. In the “CEUS-evaluation” technique, contrast-enhanced US (CEUS) is usually performed before and immediately after US-guided percutaneous ablation, and the lesion is considered completely ablated when the defected area completely covered exceeds the edge of the primary lesion by 1–2 mm, while the surrounding thyroid parenchyma was filling by contrast agent (36, 37). One month after thermal ablation, CEUS is repeated to determine whether the nodule area has shrunk. In one study, 1-year after RFA, CEUS was repeated to examine whether the area remained as a small scar-like lesion (18)

## Ultrasound-Guided Thermal Ablation of Benign Thyroid Nodules

Ultrasound (US)-guided thermal ablation has been reported to significantly improve nodule-related symptoms and cosmetic issues by reducing the volume of benign nodules (30). Thermal ablation is generally used in the treatment and management of cytologically proven benign nodules (2, 4, 9, 12, 38–41), and RFA, MWA, and LA have similar beneficial therapeutic effects. The limitations of ultrasound-guided thermal ablation of benign thyroid nodules mainly including heat sink effect, relies on a high level of experienced clinicians and intracystic hemorrhage.

### Clinical Applications of Ultrasound-Guided Thermal Ablation of Benign Nodules and Progress

Over the past few years, thermal ablation techniques have been shown to be capable of reducing thyroid nodule volume to improve nodule-related pressure symptoms and cosmetic issues (2, 30, 42, 43), and is regarded as a rapid outpatient procedure (44). For example, A systematic review and meta-analysis comprising 9 studies involving 1461 patients with 1845 benign thyroid nodules had a pooled volume reduction rate (VRR) of 54.3%, 73.5%, and 88.6% at 3, 6, and 12 months after US-guided percutaneous MWA, respectively (1). Another study examined 46 patients with 50 nodules that were treated with RFA; the mean volume reduction was 53 ± 14.9% after 1 month (p < 0.0001) and 67 ± 17.6% (p <0.0001) after 6 months of follow up (30). Furthermore, a long-term follow-up study indicated that LA was effective at the final evaluation, a median reduction of the total nodule volume of 92% was observed, and remission of the cystic part (volume ≤1 mL) was reported in 82 of 110 (75%) patients (45). RFA was found to be superior to LA in terms of the pooled VRR and the number of patients who underwent delayed surgery in a subgroup analysis (46). A systematic review and meta-analysis of 963 papers included 12 studies on RFA and 12 studies on LA, assessing 1186 and 2009 benign non-functioning solid thyroid nodules, respectively. VRR at 6, 12, 24, and 36 months was 60%, 66%, 62%, and 53%, respectively. RFA VRR was 68%, 75%, and 87%, respectively. LA VRRs were 48%, 52%, 45%, and 44%, respectively. Nodules smaller than 30 ml performed better than larger ones (47). It is worth noting that with the continuous progress of ablation technology and the extension of the development time of this technology, there has been significant improvement in the therapeutic success rate of thermal ablation in the treatment of benign thyroid nodules over the past few years. In one study, the therapeutic success rate after 6 months and 1 year were found to be 81.8% and 90.9%, respectively, after treatment with RFA (17). More recent studies have reported therapeutic success rates of 97.8% and 95.45% after thermal ablation (30, 48).

Thermal ablation not only reduces the volume of thyroid nodules but it also reduces postoperative complications and protects thyroid function to the greatest extent possible to providing a better prognosis (49). Seven studies involving 1289 patients were included in another systematic and meta-analysis to compare the safety and efficacy of US-guided thermal ablation and conventional thyroidectomy for benign thyroid nodules (50). The results showed that in the thermal ablation group, the incidences of hoarseness, hypothyroidism, and postoperative pain were lower, the hospitalization time was shorter, and the postoperative cosmetic effects were better than in the conventional thyroidectomy group (50). Another meta-analysis of data from 1090 patients with 1406 benign thyroid nodules in 20 articles found that there was a decrease in largest diameter, symptom score, cosmetic score, thyrotropin, triiodothyronine, free thyroxine level, and vascularity after RFA, with an unchanged free thyroxine level and an increased thyrotropin level (51).According to another systematic review and meta-analysis, 411 autonomous functioning thyroid nodules were treated by thermal ablation in 391 patients across 14 studies. At a mean follow-up period of 12.8 months, 71.2% of patients had their thyroid-stimulating hormone (TSH) levels normalized. During follow-up, no patients developed hypothyroidism or a life-threatening complication (52).

### Limitations of Ultrasound-Guided Thermal Ablation of Benign Thyroid Nodules

The effectiveness of thermal ablation can be inhibited by the heat sink effect, which is characterized by the presence of nearby vessels ≥3 mm in diameter, which can dissipate heat away from target tissues (7). In addition, thermal ablation can sometimes be challenging, and demands a higher level of experience (13, 53). Further, US-guided percutaneous thermal ablation can lead to intracystic hemorrhage when used in the treatment and management of cystic thyroid nodules, which significantly prolongs ablation time and can negatively affect treatment outcome. Dong et al. (34) reported an incidence of 10.3% (9/87 cysts) of intracystic hemorrhage, and larger cystic thyroid nodules were more likely to develop intracystic bleeding during US-guided percutaneous MWA. The efficacy of US-guided percutaneous LA is affected by thyroid nodule morphology (54). Thermal ablation had a complication rate of about 3.8%, which was acceptable, have also been reported. Nodule rupture associated with breakdown of the anterior thyroid capsule and formation of fluid collection in the anterior neck, which can require surgical drainage, is one of the most common complications of thermal ablation (16, 21). Additionally, recurrence and regrowth have also been reported in some cases. Negro et al. (55) performed a retrospective study to evaluate patients with benign solid thyroid nodules who were treated with LA and followed up for 5 years. Of the 104 participants included in that study, 31 (29.8%) had a VRR <50.0% after 12 months, and nodule regrowth was observed in 39 (37.5%) patients. Nodule regrowth and recurrence had an inverse relationship with the 12-month VRR; that is, a small VRR after 12 months was associated with a higher risk of regrowth (*p <*0.001). Non-spongiform composition was also reported to increase the risk of regrowth (*p <*0.001).

## Progress and Challenges In The Clinical Applications Of Ultrasound-guided Thermal Ablation In Malignant Thyroid Nodules

An estimated 7–15% of thyroid nodules are cancerous. There are various types of thyroid cancer, the most common type is PTC (56, 57). Over the past few years, PTC have been diagnosed more frequently due to the advancement in medical imaging technology, resulting in a significant increase in the incidence of papillary thyroid micro carcinoma (PTMC). US-guided thermal ablation for minimally invasive treatment has been employed in the treatment and management of PTC, medullary thyroid cancers (MTC) (36, 40, 58, 59), without any evidence of extraglandular spread or nodal metastases and recurrent malignant thyroid nodules. Thermal ablation of malignant nodules has some advantages over surgery, but it is very important to evaluate the nodules type and choose appropriate nodules that is candidate for thermal ablation or surgery. Ultrasound-guided thermal ablation in malignant thyroid nodules also has some challenges.

### Progress in the Clinical Applications of Ultrasound-Guided Thermal Ablation in Malignant Thyroid Nodules

In recent years, relevant research has proved that RFA, MWA, and LA have similar beneficial therapeutic effects in malignant Thyroid nodules especially in PTMC or low-risk small PTC who are not eligible for surgery with very low complication rates (12, 19, 36). Ji et al. (36) performed a study on 37 patients with PTMC who underwent US-guided percutaneous LA. Measurement of lesion volume, serum thyroid hormone levels, and clinical evaluation were performed at 1, 3, 6, and 12 months, with a follow-up every 6 months. The authors reported that all patients were successfully treated with percutaneous LA without serious complications: 12/37 (32.4%) primary lesions had disappeared, and 24/37 (64.9%) remained as cicatricial hyperplasia at the last follow-up. Yue et al. (60) assessed the longer-term effectiveness of US-guided MWA, and their study included 119 patients with unifocal PTMC without clinically cervical or distant metastasis. They found a significant reduction in tumor volume immediately after MWA, from 1.87 ± 1.03 mL to 0.01 ± 0.04 mL at the final evaluation (*p <*0.001). The mean VRR was 99.4 ± 2.2%, and 107 cases (93.9%) had complete remission. In a long-term study consisting of 21 nodules from 15 patients with PTMC, the treatment was successful for all nodules, without any occurrence of severe complications, with 20/21 nodules completely absorbed at the final follow-up after percutaneous MWA. No recurrence was noted in of the 15 patients at 3-year follow-up (61). US-guided percutaneous RFA has also been shown to be effective in the treatment of low-risk small PTCs (62). Cao et al. (12) performed a study with 172 patients (38 men and 134 women) with solitary T1bN0M0 PTC. They observed significantly smaller diameters and volumes of ablation zones at 6, 12, 24, 36, and 48 months after treatment (*p <*0.05), and there was complete disappearance of the tumors (61.6%) at the final follow-up.

It is worth noting that in recent years, with the application of thermal ablation technology in malignant thyroid nodules, more and more meta-analysis and systemic reviews have fully confirmed its effectiveness and safety. A systematic review and meta-analysis included ten eligible studies with a total sample size of 270 patients and 415 thyroid nodules. The pooled proportion of VRR after RFA was ≥50%, the pooled proportion of complete disappearance after RFA was 68.8%, and the pooled proportion of recurrence after RFA was 0.0% (63). Another study used a total of 12 eligible studies, with a sample size of 1187 patients and 1284 PTMCs. In this systematic review and meta-analysis, RFA, MWA, and LA all showed a significant reduction in PTMC tumor volume (p< 0.05) (64). Cui et al. (65) found that seven studies met the inclusion criteria for their meta-analysis, which included 33 original articles. There were five retrospective studies, two prospective trials, one controlled study, and one multi-center study among them. Between the baseline and final follow-up visits, there were significant improvements in nodule volume, clinical symptom scores, and beauty scores. The pooled proportions of complete disappearance and recurrence, as well as the pooled estimates of mean volume reduction and its rate of the treated PTMC, were assessed using random-effects modelling (65). In another systematic review and meta-analysis involving 11 eligible papers and 715 patients (66). The proportions of complete disappearance and recurrence of PTMC were 57.6% and 0.4%, respectively, in a pooled analysis and the mean volume reduction and rate were estimated to be 73.5 mm^3^ and 98.1%, respectively (66).

Collectively, all these studies suggest that thermal ablation is useful, effective, and safe for the treatment of malignant thyroid nodules, and can serve as an alternative to the traditional surgical methods. At the same time, the consistency in these studies could be as a result of the ablation procedure that was employed in this studies. Thermal ablation techniques such LA, MWA and RFA have a similar technical procedure and the factors that could affect the ablation procedure is similar. Although the current studies have shown that thermal ablation is effective and safe when used in the treatment of malignant thyroid nodules, long-term studies for at least 10–15 years are crucial to better observe local recurrence, lymph node metastases, and distant metastases.

Recently, there has been some attempts about appliance of thermal ablation in malignant thyroid nodules other than PTC. Biamonte et al. (40) for the first time reported the use of RFA in a patient with non-metastatic MTC who was not eligible for surgery because of high anesthesiological risk. In their case report, the use of three transisthmic insertions of a RF electrode with moving shot technique was adopted, neck ultrasound showed a complete necrosis of the tumor. After 6 and 12 months of follow-up, US examination revealed fibrotic tissue in place of the thyroid nodule, without evidence of cervical lymph-node metastases. Tong et al. (62) also reported the use of thermal ablation technique in the treatment of recurrent MTC with cervical lymph node metastases. Their case report included the case of a 56-year-old woman with cervical lymph node metastases of MTC. Neck US and a contrast-enhanced US showed obvious shrinkage in the ablation zones four months after treatment with thermal ablation. However, further controlled-studies are needed to validate thermal ablation role in the management of MTC.

Recently, there has been some new progress about appliance of thermal ablation in T1 staging of PTC. Most studies have focused on thermal ablation of T1N0M0 stage tumors. Such as the short-term outcome of T1bN0M0 PTC after US-guided RFA was evaluated in a retrospective study, in the study, 89 patients with T1bN0M0 PTC were treated with RFA guided by US. The average period of follow-up was 18.87.3 months. During follow-up, the ablation zones gradually decreased, with 38 (42.7%) of them totally disappearing, 30 months following ablation, the VRR was (99.2223%), during follow-up, two patients (2.2%) experienced tumor recurrence, and one patient (1.1%) experienced cervical lymph node metastases (67). Another study also did a multicenter retrospective study of 847 patients (660 females) who underwent thermal ablation for PTC (673 T1a, 174 T1b), 645 were subjected to MWA, while 202 were subjected to RFA, the technical success rate was 100%, the maximum diameter and volume of the ablation zone increased during the first month after ablation (P<.001), but there was no difference by the third month; subsequently, the tumors shrank in size at 6, 9, and 12 months (all P<.001). Tumors completely disappeared in 68% of patients (68). The authors all concluded that thermal ablation is an effective and safe therapeutic option for patients with single T1N0M0 PTC, according to this multicenter study. The majority of studies on thermal ablation of malignant thyroid nodules in literature focus on tumors in the T1N0M0 stage. Because patients are concerned about incomplete tumor ablation or local recurrence when the tumor’s maximal diameter is bigger than 2 cm, or when the tumor is in the T2N0M0 stage, they frequently choose surgical treatment. As a result, the relevant research literature is limited. Only few cases choose thermal ablation, particularly for those who are surgically ineligible. The efficacy and safety of US -guided RFA for treating T2N0M0 PTC were investigated, twelve individuals with T2N0M0 PTC were enrolled in this investigation (5 men and 7 women), and all of the tumors were ablated according to the plan (69). The average duration of follow-up was 24.1± 6.9 months (range, 13-33 months), at the last follow-up, the tumor volume had decreased significantly from 4.4 ± 2.8 ml to 0.3± 0.5 ml, and the VRR was 93.7± 7.6%, with two tumors (16.7%) disappearing, during follow-up, no new or recurrent tumors were discovered, and no local or distant metastasis was discovered (69).

Recently, there has been some new progress in ultrasound-guided thermal ablation of cervical lymph node metastasis. In a few cases, lymph nodule metastasis can occur in some patients after treatment with US-guided percutaneous thermal ablation or after surgery. US-guided thermal ablation has also been shown to be effective in the management and control of cervical lymph node metastasis. Twelve months after treatment of nodules of metastatic PTC lymph nodes using single-fiber LA, mean baseline volumes reduced from 0.29 ± 0.12 to 0.03 ± 0.03 mL, and the mean VRR was 90.3 ± 7.6%. These findings indicate that thermal ablation, such as LA performed under US guidance, is promising and favorable for the treatment and management of metastatic lymph nodes in PTC, with appropriate ablation modes (34). Han et al. (70) evaluated the safety and efficacy of percutaneous US-guided microwave ablation for 98 cervical metastatic lymph nodes in patients with PTC. After a single session, all 98 metastatic lymph nodes had been successfully treated and 100% ablation was observed. There was a reduction in the average longest diameter and shortest diameter of the tumors, from 13.21 ± 5.86 mm to 6.74 ± 5.66 mm and from 9.29 ± 4.09 mm to 4.31 ± 3.56 mm at the final follow-up respectively. Recurrence and regrowth at the ablated sites were not observed. In another study, Teng et al. (71) observed that all the treated lymph nodes were absorbed within 18 months after percutaneous microwave ablation and, after a mean follow-up period of 32 months (range: 18–42), no new recurrent lesions were observed. These studies indicate that the treatment of cervical metastatic lymph nodes in PTC with low-power microwave ablation is safe, promising, and efficient.

### Clinical Applications of Surgical and Ultrasound-Guided Thermal Ablation in Malignant Thyroid Nodules Comparison

Thermal ablation of malignant nodules has some advantages over surgery. Recent studies have shown that lidocaine local anesthesia is used for thermal ablation, rather than general anesthesia for traditional resection. Thermal ablation can be performed in the form of outpatient surgery, it has less impact on vital signs, and has lower systemic stress response than surgical patients. Thyroid mass ablation is usually done under ultrasound guidance, which helps to accurately locate the lesion to be ablated and precisely control the power, time and range of the ablation needle, and preserve normal thyroid tissue, as well as to protect normal surrounding tissues while preventing postoperative adhesions under effective fluid isolation conditions, with little damage to thyroid function, the serum FT3, FT4, and TSH levels only slightly change afterwards, and there is no long-term use of levothyroxine after ablation, so patients gets a good health related quality life, and they spend less amount of money on the cost of treatment (72–77). Recent comparative studies have also shown that there is less operation time as well as short hospitalization time for a patient undergoing thermal ablation for malignant nodules compared to surgery, because there is less trauma to the body, less incidence of complications, there is also a good therapeutic effect. Zhou et al. (78) in their retrospective study on ultrasound-guided laser ablation versus surgery for solitary papillary thyroid microcarcinoma reported mean hospital stay and procedure time in the LA group were shorter than those in the surgical group. The pain score of patients after thermal ablation is lower than that of surgery.

The main disadvantage of thermal ablation of malignant nodules compared to surgery is the incomplete response and local regrowth which leads to repeated ablation or surgery (3). So, it is very important to evaluate the nodule type and choose appropriate nodule that is candidate for thermal ablation or surgery. Especially, for substernal nodule, and deep located malignant thyroid nodules, thermal ablation is not appropriate. The moving-shot or fixed applicator technique as already stated is used based on tumor characteristics. For benign thyroid nodules, both technique could be employed, however for malignant nodules the moving shot techniques is recommended and has been employed in many studies (12, 60). Most malignant nodules have irregular shape and could be closer to critical structures, so prolonged fixation of the electrode for complete ablation could be dangerous to surrounding critical structures (61).Thermal ablation of malignant nodules needs a careful evaluation of the surrounding structure and hydrodissection can be performed to displace other structures close to the tumor and has been employed in some studies involving malignant nodules (19, 70).

It is noteworthy that, thermal ablation may not be inferior to surgery oncologically, and it is a relatively safe and cost-effective treatment option for PTMC and single T1N0M0 stage PTC. Seven retrospective studies with a total of 867 patients were included in a systematic review and meta-analysis (79). Thermal ablation was found to reduce complication with a pooled odd ratio of 0.24(95% CI 0.13 to 0.43), postoperative length of stay with a pooled standardized mean difference of -3.14(95% CI -4.77 to -1.51), and cost during the perioperative period with a pooled standardized mean difference of -1.69(95% CI -3.18 to -0.20), according to the study (79). Also, researchers compared the efficacy and safety of RFA to surgery in patients with T1bN0M0 PTC of various ages. A total of 204 patients with an isolated, solitary, intrathyroidal T1bN0M0 PTC who underwent either RFA (n=94) or surgery (n=110) were enrolled retrospectively and divided into two age groups (<45 years, ≧45 years), there were no significant differences in the treatment groups in terms of age, gender, or tumor size (80). While the RFA group had lower costs and a significantly shorter operative duration than the surgery group, no significant differences in tumor progression or complications were observed, in the surgery group, older patients had higher hospital charges than younger counterparts, but this was not the case in the RFA group. RFA had a similar prognosis to surgery, but was related with reduced total cost in both young (<45 years) and middle-aged (≧45 years) patients with T1bN0M0 PTC, according to the authors. As a result, RFA could be a viable and safe alternative to surgery for patients with T1bN0M0 PTC (80).

### Recurrence and Metastatic Situations of Ultrasound-Guided Thermal Ablation in Malignant Thyroid Nodules

There has been remarkable progress in terms of tumor recurrence, lymph node metastasis, and distant metastasis after thermal ablation. In a few cases, lymph nodule metastasis can occur in some patients after treatment with US-guided percutaneous thermal ablation. Xiao et al. (40) reported a complication rate of 1.5% of lymph node metastases after treatment of patients with T1b PTC using percutaneous US-guided RFA, during a mean follow-up period of 20.5 months. Instead of totally dissecting lesions from patients, the goal of thermal ablation is to achieve tumor coagulative necrosis. As a result, the risk of partial ablation and local recurrence exists. In addition, insufficient tumor necrosis may increase patients’ worry and fears about recurrence and lymph node metastases (81). In their case report, Ma et al. (82) examined a multi-center medical database and enlisted 12 patients who had previously been treated with thermal ablation and were now receiving surgical therapy. All of the cases had incomplete ablation, and 66.7% of them had lymph node metastases, according to the postoperative pathology results. Thermal ablation as a treatment for operable patients with PTC should be recommended with care, and it is very necessary to pursue complete ablation of malignant nodules because incomplete ablation of malignant nodules may promote local recurrence and distant metastasis, according to the conclusions of this study (82). LA has been reported to have a higher recurrence rate than MWA and RFA (78, 83). The cause of this phenomenon was unknown, but lower power output and different operating procedures in these two ablation methods could be factors (3).

But some long-term follow-up studies recently indicated that thermal ablation of primary PTMC resulted in no local recurrence, lymph node metastasis, or distant metastasis (46, 83–85). In their prospective study for treating microcarcinoma with RFA, Zhang et al. (86) reported complete tumor disappearance. During the follow-up period, no local tumor recurrence or metastasis to lymph nodes in the neck was observed in their study. Initial complete ablation is more important than volume rate reduction and lesion disappearance during follow-up because it directly represents a successful operation procedure and low risk of residual *in situ* after an operation in lesions (87). Several studies have found that PTMC patients who received thermal ablation therapy have a low recurrence rate. In a 4-year follow-up trial, Kim et al. (61) found that the mean VRR was 98.5% and the total disappearance rate was 66.7%, with no local tumor recurrence or lymph node metastases in the neck. In a retrospective study of 414 patients, the overall incidence of local tumor advancement following RFA was only 3.62%, including lymph node metastasis (0.97%) and recurrence PTMC (2.42%) (88).

## Discussion

Owing to the complications associated with surgery, patients usually refrain from this procedure. In recent years, clinicians have therefore adopted the use of minimally invasive techniques to treat both benign and malignant thyroid nodules. Thermal ablation of benign thyroid nodules and malignant nodules have achieved significant success in thyroid nodule volume reduction, safety, efficacy, and cosmetic issues. The key reasons that patients usually opt for treatment of benign thyroid nodules are cosmetic-related issues and pressure symptoms due to nodular volume, and thermal ablation have proven to be successful in addressing these problems (2, 15, 30, 49, 89). According to literature, the complication rate and adverse effects of ablation techniques are less, compared to traditional methods—surgery.

Minimally-invasive interventions such as LA, MWA, and RFA have been shown to be effective for nodules that are predominantly solid or solid and mixed (1, 11, 25, 28, 30, 31, 38, 44, 89, 90). In addition, there have been more studies on the use of LA for the treatment of malignant thyroid nodules than on the use of RFA and MWA. Thermal ablation of malignant thyroid nodules produces a higher VRR than that used for the treatment of benign thyroid nodules (3). Since there is a significant correlation between the energy delivered per mL of nodule tissue and the percent volume decrease of thyroid nodules, it is necessary to pay more attention to the energy delivered during the ablation process to achieve the desired results (42). It is known that larger size, the delivery of lower energy, and incomplete and insufficient ablation of the external border of the nodules has an association with regrowth rates (55, 91). Consistent with this, Wang et al. (92) showed that larger initial volume, more irregular blood vessel, and nodules adjacent to the critical structures were found in the recurrence group.

The use of ablation techniques in the treatment of malignant thyroid nodules in PTC is a little controversial because, PTC is a slow-growing disease, and local recurrence, lymph node metastasis, and distant metastases can occur after treatment with ablation, it’s critical to assess the completeness of TA for treating PTC in the initial postoperative evaluation to limit the likelihood of residual lesions and LNM (61). Some researchers believe it has the potential to not only achieve the ultimate goal of treating PTC, but also to assist patients cope with the psychological stress that comes with a cancer diagnosis, current studies have shown that thermal ablation is feasible and effective when used in the treatment of malignant thyroid nodules of PTC, with very low complication rates (12, 33, 40, 58, 60, 61). Larger, longer studies should be encouraged to address PTC recurrence and multifocality. The regeneration of a certain number of nodules, which necessitates additional TA operations or surgery, is a significant disadvantage of using TA. There are currently no long-term randomized controlled trials comparing the cost/effectiveness of TA vs surgery. A proactive strategy to prevent TA failure should include the selection of the nodule, the optimization of interventional procedures, and, finally, a well-timed second treatment. When it comes to the use of TA in the treatment of malignant nodules like PTC, there are still numerous gaps in the research that must be filled. To begin with, the majority of clinical studies have used a single-center retrospective cohort study design, which is prone to recall bias. The majority of available experimental data on the efficacy and safety of TA came from Asia, particularly China. As a result, it’s yet uncertain whether these strategies will work equally well in populations of various races and places (87).

Thermal ablation techniques such as ultrasound guided percutaneous LA is capable of unveiling thyroid antigen which can induce autoimmunity in patients. It is very necessary to check for thyroid antibodies before and after percutaneous laser ablation. Even though there has been some studies on effects of thermal ablation such as laser ablation on the immune function, these studies were mostly short term studies, there is therefore the need for long term follow-up and prospective studies to monitor the thyroid antibodies and as well monitor the occurrence of autoimmunity in patients treated with percutaneous thermal ablation such as laser ablation.

It is worth noting that low-power ablation strategies can achieve better tumor absorption and volume reduction rates than high-power ablation strategies. This is because moderate tumor coagulative necrosis is more easily absorbed than burns in the ablation area. The reason is that, it is more conducive to the infiltration of immune cells and the release of inactivated tumor antigens, more conducive to the absorption of the tumor after ablation, thereby stimulating the body to produce anti-tumor immunity. However, whether it will promote tumor recurrence and distant metastasis remains to be further studied (58, 61).

## Conclusion

In the treatment of benign thyroid nodules, ultrasound-guided thermal ablations including LA, MWA, and RFA are well-established minimally invasive treatments that can be regarded viable alternatives to surgery. Although there is still limited evidence in patients with thyroid cancer, the findings in terms of local tumor management are encouraging.

## Author Contributions

EA, J-hF, and F-JX contributed equally to this study. EA, J-hF, and X-QQ contributed to the conception and design of the study. EA wrote the first draft of the manuscript. F-JX, Y-ZR, DA, QC, XW, and YW wrote sections of the manuscript. All authors contributed to the article and approved the submitted version.

## Funding

This study was financially supported by National Natural Science Foundation of China (Project No.: 81771848, 81971629) and Zhenjiang Commission of Science and Technology (Project No. SH2020046).

## Conflict of Interest

The authors declare that the research was conducted in the absence of any commercial or financial relationships that could be construed as a potential conflict of interest.

## Publisher’s Note

All claims expressed in this article are solely those of the authors and do not necessarily represent those of their affiliated organizations, or those of the publisher, the editors and the reviewers. Any product that may be evaluated in this article, or claim that may be made by its manufacturer, is not guaranteed or endorsed by the publisher.
